# Household and community drivers of *Aedes aegypti* infestation in tropical cities: Guiding community-led interventions

**DOI:** 10.1371/journal.pntd.0014728

**Published:** 2026-10-01

**Authors:** Eimear Griffin, Thiago da Mata Barreto, Zoe Ward, Juliet Oliveira Santana, Hussein Khalil, Mike Begon, Mitermayer Galvão G. Reis, Cleber Cremonese, Federico Costa, Hernan D. Argibay, Guy Howard

**Affiliations:** 1 School of Civil, Aerospace and Design Engineering, University of Bristol, Bristol, United Kingdom; 2 Cabot Institute for the Environment, University of Bristol, Bristol, United Kingdom; 3 Institute of Collective Health, Federal University of Bahia, Salvador, Bahia, Brazil; 4 Gonçalo Moniz Institute, Oswaldo Cruz Foundation, Ministry of Health of Brazil, Salvador, Bahia, Brazil; 5 Department of Evolution, Ecology and Behaviour, The University of Liverpool, Liverpool, United Kingdom; 6 Faculty of Medicine of Bahia, Federal University of Bahia, Salvador, Bahia, Brazil; 7 Department of Epidemiology of Microbial Diseases, Yale School of Public Health, New Haven, Connecticut, United States of America; 8 Fiocruz Rondônia, Oswaldo Cruz Foundation, Ministry of Health of Brazil, Porto Velho, Rondônia, Brazil; Institut Pasteur du Cambodge, CAMBODIA

## Abstract

Disadvantaged communities characterised by poor sanitation conditions, overcrowding, and limited vector control are highly susceptible to *Aedes aegypti* proliferation and arboviral disease transmission. Effective mosquito vector control requires a detailed understanding of *Ae. aegypti* oviposition preferences and spatial distribution within such urban environments. This study aimed to identify key environmental and behavioural drivers of *Ae. aegypti* proliferation in disadvantaged communities of Salvador, Brazil, combining household surveys, entomological assessments, and remote sensing to guide targeted public health interventions. Between March and December 2023, using a systematic sampling approach, an entomological survey was conducted in 174 households, alongside a socio-demographic and environmental visual inspection survey in 119 households within two disadvantaged communities of Salvador. Ovitraps were used to collect *Ae. aegypti* eggs and immatures, providing a detailed assessment of mosquito density. The visual inspection survey identified potential breeding sites (sources of standing water) for *Ae. aegypti* in the peri-domestic environment. Landcover classification was performed to provide insights into vegetation coverage and other landscape features in the study area. Multivariate modelling explored associations between environmental and household factors, and *Ae. aegypti* density. Of the 303 recovered ovitraps, 85 (55.6%) indoor and 123 (82.0%) outdoor were positive for eggs, with a total of 11,211 *Ae. aegypti* eggs recorded. Environmental factors strongly associated with egg abundance included ovitrap location, garbage deposited in front of the house, proximity (within 10 m) to an open sewer, and the presence of open plastic containers in the peri-domestic environment. No significant spatial clustering of mosquito egg density was observed. Breeding was most prolific in the peri-domestic environment, highlighting the importance of keeping these areas clean and well-managed. Improving waste disposal, water storage practices, and sanitation infrastructure could enhance the effectiveness of vector control. These findings provide evidence to inform integrated strategies for reducing arboviral disease risk in disadvantaged urban settings.

## Author summary

Mosquito-borne diseases are a major threat to human health, particularly in disadvantaged communities that lack the resources to manage them. *Aedes aegypti* mosquitoes, which transmit dengue, Zika, and chikungunya, thrive in tropical climates and are extending their range as temperatures increase due to climate change. Therefore, it is important to develop effective strategies to control these mosquitoes to minimise the spread of disease. Environmental management is considered a sustainable way of reducing mosquito breeding sites. This study identified household and community drivers of *Aedes aegypti* mosquitoes in vulnerable communities of Salvador, Brazil.

Through household surveys and environmental inspections, information on water storage, waste management, and sanitation was recorded. In addition, mosquito eggs were collected and counted to see how these environmental conditions influenced actual mosquito infestation. The household and community characteristics that were linked to higher mosquito counts were the presence of open plastic containers that can hold stagnant water, accumulated garbage near homes, and living close to an open sewer. By identifying these drivers of mosquito breeding, our work can help guide more targeted mosquito-control efforts. This means that communities can design practical solutions that maximise the use of resources to reduce mosquito breeding and protect residents’ health.

## Introduction

The burden of vector-borne diseases is rising rapidly, posing an increasing threat to public health worldwide. Diseases such as dengue, Zika, chikungunya and yellow fever, all primarily transmitted by the *Aedes aegypti* mosquito in urban environments, have shown significant increases in incidence in recent years. For instance, the World Health Organisation (WHO), currently estimates that there are approximately 390 million new dengue virus (DENV) infections per year, of which 96 million are symptomatic [1]. These mosquito-borne diseases place significant health and economic stress on affected regions, particularly low- and middle-income countries (LMICs), by draining resources and overwhelming health-care systems [2].

Tropical regions characterised by high average temperatures, humidity, and annual rainfall are the most suitable for *Aedes* mosquitoes [3]. However, the *Ae. aegypti* mosquito is currently extending its range due to climate change, meaning previously unexposed populations are now at risk [4]. Although there is extensive knowledge about the *Ae.aegypti* mosquito, poor practices, inadequate infrastructure, and other socioenvironmental determinants have limited the effectiveness of vector control actions thus far, thereby increasing the risk of arboviral disease [5].

*Aedes aegypti* is widely distributed across Brazil, and according to the WHO, it is the country that reports the highest number of dengue cases in the world. Approximately 10 million cases were reported between 2010–2019 [6,7]. Salvador, the capital city of the state of Bahia, is a densely populated urban environment with an estimated population size of 2.5 million, a significant proportion of its inhabitants live in marginalized communities, commonly referred to as favelas [8]. It has a tropical climate consisting of warm temperatures and high levels of precipitation, with the average daily temperature varying between 24 and 32°C throughout the year. These climatic conditions, combined with high population density, favour mosquito proliferation; therefore, Salvador is considered a high-risk area for arboviral disease transmission [9].

In Salvador, it is the urban poor residing in disadvantaged communities that are most vulnerable to arboviral diseases due to poor sanitation, insufficient urban infrastructure, and lack of access to healthcare [6,10]. Furthermore, crowding in these communities provides more feeding opportunities for mosquito vectors and greater interaction with human hosts, thereby increasing the risk of virus transmission [6].

Rapid urbanisation has created more suitable breeding sites for *Aedes* mosquitoes. *Ae. aegypti* has become well-adapted to urban environments, utilizing artificial containers that accumulate water as breeding sites, such as tyres, tanks, buckets, bottles and pots [11]. Female adult *Aedes* mosquitoes lay their eggs just above the waterline in shallow pools of stagnant water, which hatch when immersed in water. However, the eggs can survive in dry conditions and resist desiccation for months before this happens, increasing survival rates and breeding opportunities [12,13]. Larvae and pupae undergo maturation in aquatic environments; therefore, water quality, including the level of dissolved solids and oxygen, has an impact on larval density [14–16]. *Aedes* mosquitoes have been found to exhibit “skip oviposition” behaviour due to larval competition, spreading eggs across different containers during a single gonotrophic cycle [17,18].

Environmental sanitation conditions are often poor in disadvantaged urban communities, leading to increased disease risk. This is due to a range of factors such as limited access to resources, lack of education and awareness, overcrowding, inadequate infrastructure, and vulnerability to natural disasters [19,20]. Water supply systems are often inadequate in these communities, which can promote improvised water storage. This increases the amount of stagnant water in domestic environments and creates breeding habitats for *Aedes* mosquitoes, thereby increasing the risk of arboviral transmission. In addition, wastewater management and drainage systems are often ineffective in these communities, which can create pools of water during and after rainfall. This accumulated water can persist for an extended period, providing suitable locations for *Ae. aegypti* to lay eggs and complete their lifecycle [21].

Environmental sanitation interventions are considered a sustainable and effective approach to control mosquito vectors by ensuring a sanitary environment [22] that reduces the breeding sites and productivity of *Ae. aegypti* [23,24]. According to the WHO, such interventions fall into three categories: environmental manipulation, environmental modification, and behavior change. Environmental manipulation involves targeting and eliminating the most productive mosquito breeding sites, such as artificial containers. Environmental modification focuses on long-term improvements to sanitation infrastructure, water supply, storage systems, and waste management to minimize standing water and potential mosquito habitats. For instance, providing reliable piped water reduces the need for water storage, removing potential breeding sites for mosquitoes. These environmental measures often depend on changes in human behavior, with community knowledge, education, and participation being critical for success. Integrating environmental sanitation interventions into comprehensive mosquito prevention programs is strongly recommended to maximize effectiveness [25].

In urban settings, understanding the spatial dispersion of *Ae. aegypti* is also an important aspect of developing targeted control strategies. Studies in Brazil, Peru and Ecuador have shown spatial clustering of *Ae. aegypti* within 20–100 m of infested households [26–28]. This fine-scale heterogeneity suggests that household-level conditions play a role in *Ae. aegypti* distribution. Therefore, identifying vector ‘hotspots’ could lead to more effective disease control programs and prevent wasting resources.

Using a multidisciplinary approach, this study combines household surveys, entomological assessments, and remote sensing to investigate how sanitation conditions, waste management practices, water-storage behaviours, and land-cover characteristics influence *Ae. aegypti* proliferation within disadvantaged communities of Salvador, Brazil, providing a novel, fine-scale perspective on the interplaying factors driving *Ae.aegypti* infestation in these settings. The study goal was to produce new evidence on how localized sanitation conditions influence *Ae. aegypti* infestation to inform both community-led and targeted public health interventions.

## Methods

### Ethics Statement

This study was approved by the Research Ethics Committee of the Institute of Collective Health/Federal University of Bahia (CEP/ISC/UFBA) and by the National Commission for Ethics in Research (CONEP) linked to the Brazilian Ministry of Health under protocol numbers CAAE n° 35405320.0.1001.5030 and 32361820.7.0000.5030. Written informed consent was obtained from an adult (> 18 years old) responsible for each household prior to participation. In addition, the University of Bristol Faculty of Engineering Research Ethics Committee also approved this study, approval code 14565.

### Study design

This was a cross-sectional study of households in two disadvantaged urban communities, Nova Sussuarana and Marechal Rondon, situated in the periphery of Salvador, as shown in Fig 1 [10,20]. The study consisted of an entomological survey by ovitraps and a visual inspection survey of the peri-domestic environments surrounding households. The households and participants involved in this study had already been recruited for two ongoing trials taking place within the communities [29,30]. The household-level and community-level environmental visual inspection surveys were carried out alongside the entomological surveys during the rainy season in Salvador, from April to August 2023.

**Fig 1:**
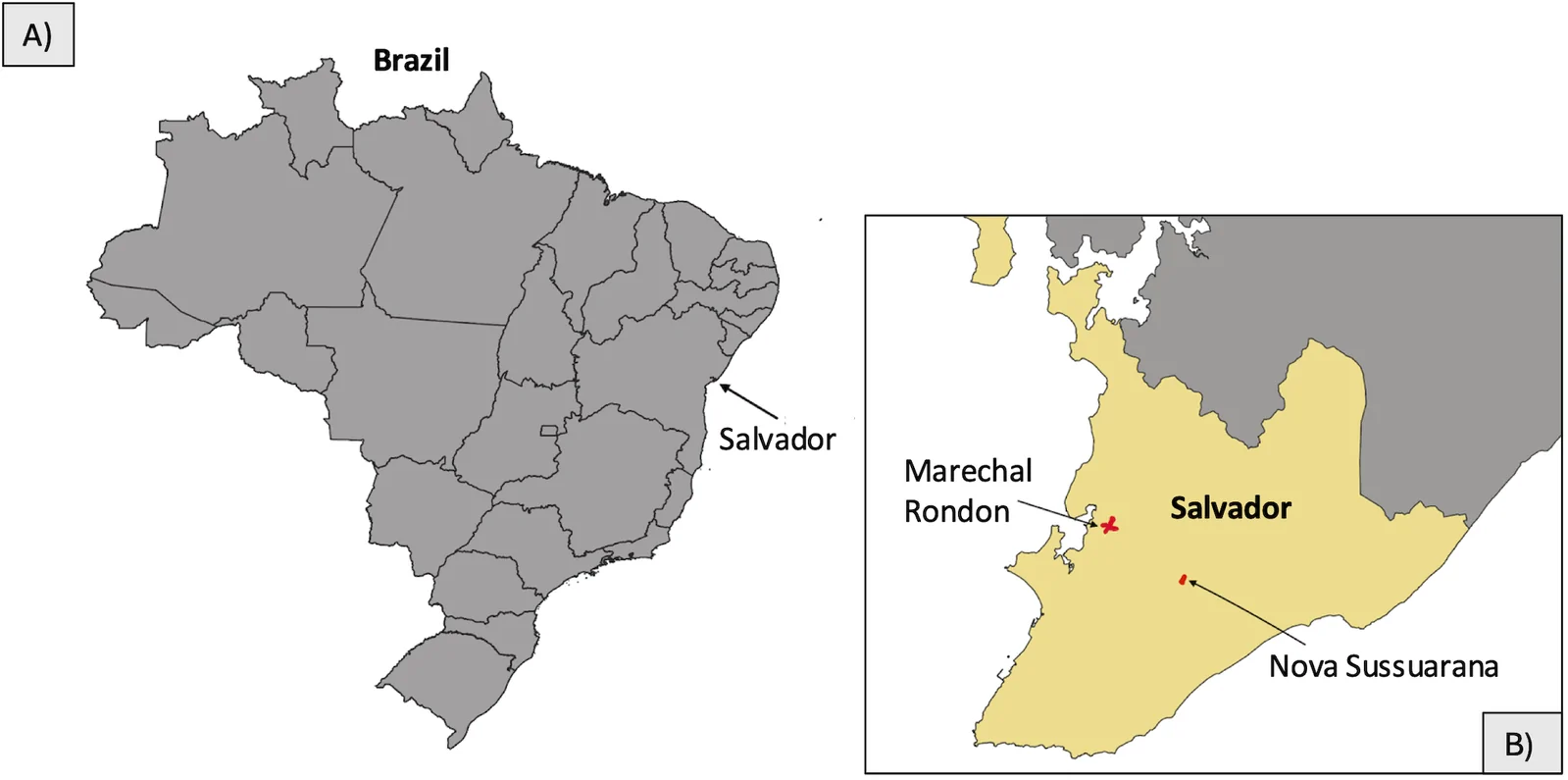
Maps delineating A) the location of Salvador in Brazil, and B) the location of Marechal Rondon and Nova Sussuarana in Salvador, Brazil. The city boundary layer was obtained from the IBGE municipal shapefile for Bahia (https://geoftp.ibge.gov.br/organizacao_do_territorio/malhas_territoriais/malhas_municipais/, public domain). Land extent was defined using OpenStreetMap land polygons (© OpenStreetMap contributors, https://www.openstreetmap.org/copyright, Open Database License (ODbL)).

Ovitrap sampling was primarily carried out during the rainy season when *Ae. aegypti* abundance is typically highest [31]. The timing was chosen to capture peak oviposition activity, allowing environmental risk factors to be assessed under conditions when the risk of mosquito-borne disease is most prevalent and targeted interventions are most needed. Although rainfall may increase the number of alternative breeding sites in the environment, the dry season presents the opposite problem; fewer natural breeding sites may increase the relative attractiveness of ovitraps, potentially leading to an overestimation of oviposition activity.

Due to violence and unrest within the communities, fieldwork had to be suspended, and the surveys were reinitiated in December 2023. A timeline of the data collection activities can be found in S1 Table.

### Study site

Nova Sussuarana and Marechal Rondon are characterised by high population density and low socioeconomic status [29,30]. Both have poor sanitation conditions with insufficient piped water supply, and inadequate waste management. While almost all the households in these communities are connected to a piped water supply service, this is unreliable, with frequent interruptions lasting hours or even days, which increases the amount of water storage by residents. There was substantial community engagement in both study sites due to previous participation in broader ongoing projects, which meant that data collection was safer and there was a greater willingness to participate [29,30]. Despite their similarities, the communities had notable structural differences. The study site in Nova Sussuarana was smaller, concentrated around a river contaminated by sewage, with dense housing backing onto the river. In contrast, the study site in Marechal Rondon was larger, and with part of the surveyed households set further away from the open sewer.

The eligible population in each study site was defined as all households within 40 metres of an open sewer or lake, as shown in Fig 2. The study site covered an area of 0.034km^2^ in Nova Sussuarana and an area of 0.093km^2^ in Marechal Rondon. In 2022, a household census was carried out that identified 413 households within the study site in Nova Sussuarana, and 775 households within the study site in Marechal Rondon. All households were geocoded during this census.

**Fig 2:**
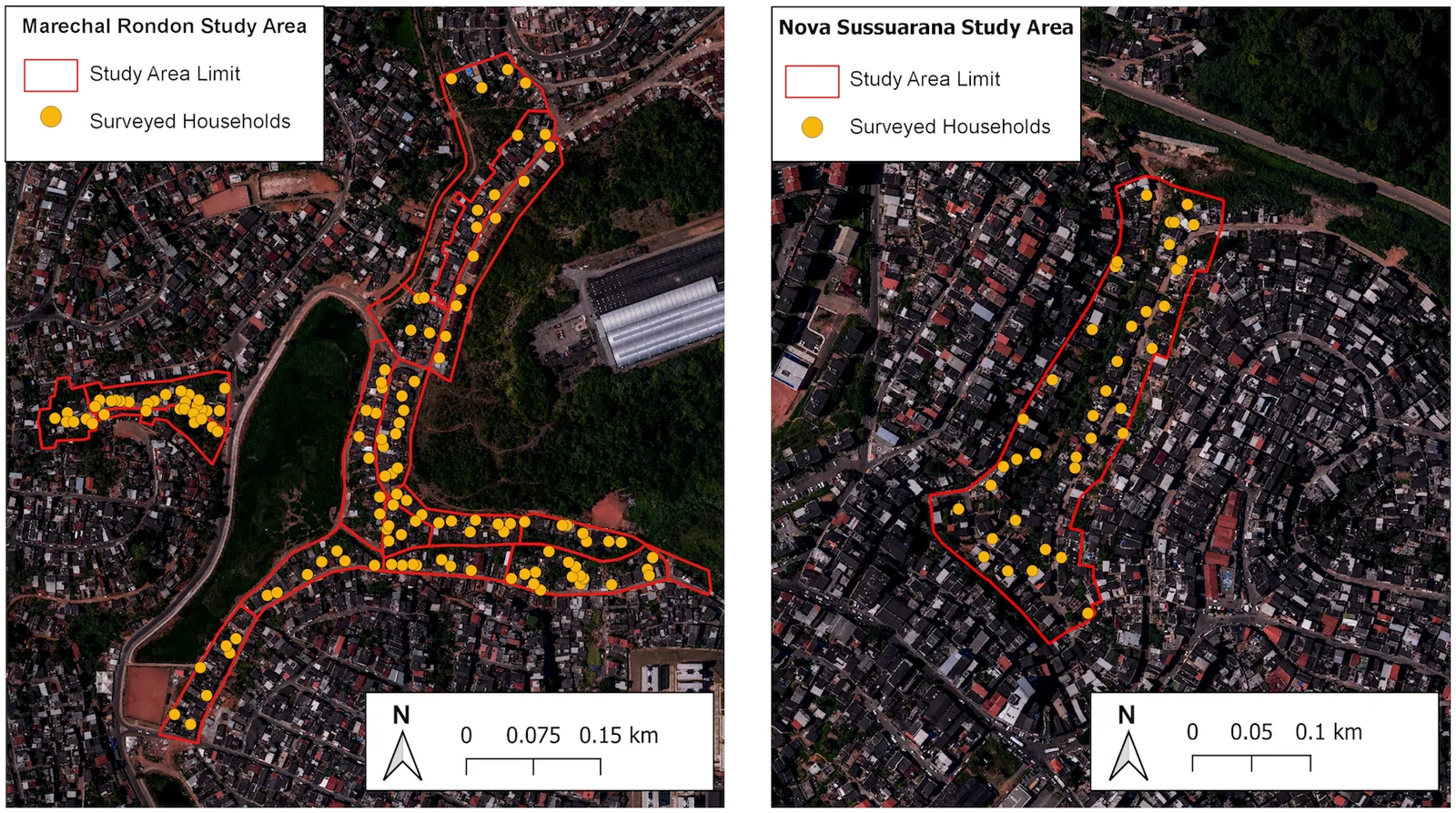
Study areas in Marechal Rondon and Nova Sussuarana showing the 174 households where ovitraps were installed. Study area boundaries provided by Salvador Municipality cartographic data. The high-resolution base orthoimage was obtained from the Salvador Municipality cartographic services via its Web Map Service available at https://www.sefaz.salvador.ba.gov.br/geosalvador/home/webmap/viewer.html?webmap=a455c391abc4495b9ce96693c4f288b6 (accessed April 2026); terms of use: https://www.sefaz.salvador.ba.gov.br/geosalvador/home/item.html?id=a455c391abc4495b9ce96693c4f288b6). The WMS is provided as publicly accessible municipal open data for consultation and reuse, subject to source attribution in accordance with the municipality’s open data and public information policies (https://www.sefaz.salvador.ba.gov.br/geosalvador/home/item.html?id=a455c391abc4495b9ce96693c4f288b6).

### Socio-demographic data collection

During the household census, all houses within the study areas were invited to participate in the socio-demographic survey. A standardised questionnaire was administered to the head of the household to gather information on socio-demographics and household practices. Relevant data was selectively extracted from this questionnaire and used as proxy indicators for household income and education levels. Data on frequency of garbage collection were also included however, as this may have changed over time, this variable was interpreted with caution. The survey tool is included in S1 Fig, and the extracted information can be found in S2 Table.

### Entomological data collection

An entomological survey was conducted in Nova Sussuarana during March-April 2023, and in Marechal Rondon during April-December 2023 to estimate the abundance and spatial distribution of *Ae. aegypti* by placing ovitraps at household locations. The ovitraps consisted of black, cylindrical plastic containers filled with 350ml water and equipped with a wooden paddle (10 cm X 4 cm) inserted and secured with adhesive tape to the edge of the container.

Ovitraps were installed in recruited households by the broader eco-epidemiological projects with an approximate density of 1 household every 312m^2^, as shown in Fig 2, aligning with the known clusters distance correlation around breeding sites for *Ae. aegypti* [26,27,32]. To achieve this, the study area was divided into 30m-sized hexagonal polygons, and then the first household available and willing to participate was selected for entomological assessment within each polygon. The ovitrap collections were conducted once in each of the recruited households.

Two ovitraps were installed per household to sample from both the intra-domestic and peri-domestic environments. The ovitraps were collected 5–7 days after installation, which corresponds to the *Ae. aegypti* lifecycle under the climatic conditions of Salvador, ensuring that the larvae remained in the aquatic stage. The ovitraps were transferred to a plastic bag, closed and labelled with the household code. Any larvae present were transferred to falcon tubes containing 70% alcohol. The wooden oviposition paddle from the ovitrap was then transported to the Laboratory of Entomology, located at Instituto Gonçalo Moniz (Fiocruz/BA) in Salvador. After field collection, paddles were kept at room temperature to allow drying and were stored until collections from each study area were completed (7–15 days after retrieval). Immature stages were not morphologically identified; instead, the presence of immatures (larvae or pupae) was recorded only as a supplementary observation during ovitrap inspection. This was not included in the analyses, given that the study focused exclusively on Egg Density Index.

The wooden oviposition paddle was then examined under a stereomicroscope (Nikon SMZ 745 T) to count and classify *Ae. aegypti* eggs using the identification keys in Consoli and Oliveira [33]. For paddle positivity and egg counts, both hatched and unhatched eggs were included. Only eggs presenting chorion melanization and intact morphology were included in the counts. Eggs that did not undergo melanization during the observation period, as well as malformed eggs, were excluded. Previous ovitrap studies conducted in Salvador in similar settlements have shown that nearly all eggs collected correspond to *Ae. aegypti,* while other species, such as *Ae. albopictus,* represent less than 1% of collections [34]. Preliminary observations from this study were consistent with these findings, with negligible representation of other *Aedes* species or mosquitoes from other genera. Based on this evidence, eggs counted on paddles were considered to be *Ae. aegypti*.

To minimise counting errors, two trained team members independently counted the eggs, and the average of the two counts was used for analyses.

In addition, monthly rainfall (mm) data corresponding to each ovitrap collection period was obtained from the Salvador automatic meteorological station, obtained from the INMET site [35], to account for temporal variation in precipitation across collection periods, as rainfall is known to influence *Ae. aegypti* abundance [36,37].

### Household-level environmental visual inspection

A visual inspection survey was carried out alongside the entomological survey in Nova Sussuarana during March-April 2023, and in Marechal Rondon during April-December 2023. The data was recorded and stored on REDCap (Research Electronic Data Capture, Vanderbilt University, Nashville, TN, USA) [38]. The visual inspection survey was performed on the same day as ovitrap installation, where access was possible. Due to violence and unrest in the communities, it was not possible to safely access all properties. As a result, visual inspections were conducted in 119 of the 174 households where ovitraps were installed, as shown in Fig 2.

The visual inspection survey was designed to identify potential environmental risk factors for *Ae. aegypti* mosquitoes in the peri-domestic environment (within a 10 m radius) surrounding each household that had ovitraps installed. The survey items were selected based on a detailed reading of previous studies outlining common breeding sites for *Ae. aegypti* mosquitoes, expert discussion, and observations of perceived vulnerabilities made during preliminary visits to the communities. The survey tool is included in S2 Fig and S3 Table.

### Community-level environmental vulnerability data collection

Data on community-level environmental risks (leaking sewers, blocked drain inlets and damaged storm drains) were collected in October 2022 in Marechal Rondon and in May 2023 in Nova Sussuarana. The spatial coordinates of points indicating environmental risks were obtained and recorded using a handheld GPS meter. The data collection for community-level environmental vulnerabilities coincided with the entomological survey in Nova Sussuarana, but there was a time-lag between these two data collection processes in Marechal Rondon. Nevertheless, since the sanitation infrastructure did not undergo any significant changes during this time period, as verified during community visits for the entomological surveys, any potential impacts on the validity of the data were deemed negligible.

The coordinates were recorded in REDCap [38] and used to calculate the proximity of each surveyed household to environmental risks. Using the Near analysis tool in ArcMap [39], the distance between each surveyed household and the nearest point of drainage vulnerability and sewerage vulnerability was calculated.

### Landcover classification

To characterise the surrounding environment of each surveyed household, a supervised classification of a Worldview-3 satellite image (July 2023; 0.31 m panchromatic, 1.24 multispectral resolution) covering the two study sites was performed using ArcMap 10.7.1 to obtain data on four landcover types: 1) water, 2) bare ground, 3) impervious surface, 4) vegetation. These cover classes were defined to reflect the dominant urban surface types in the study communities and to derive information on ecologically relevant environmental features for *Aedes aegypti* that were not captured in the ground surveys*,* such as water and vegetation [32,40]*.* A supervised classification using the Maximum Likelihood algorithm was used to enable accurate separation of vegetation and water from spectrally similar urban surfaces and shadows, which are more prone to misclassification with unsupervised approaches.

Post-classification processing was then undertaken to reduce shadow misclassification. To validate the supervised classification, randomly selected points across the raster were manually classified using ground truth data. The software classified landcover values for each point were compared to the manually assigned values using a confusion matrix.

A 30 m buffer was created around each surveyed household, based on the dispersion range of *Ae. aegypti* in urban settings [26,27], and the percentage of each landcover class within the buffer was calculated. Vegetation cover was included as a continuous variable in the analysis, based on previous evidence linking vegetation to increased *Ae. aegypti* breeding [40,41].

### Data analysis

Data was stored on RedCap (Research Electronic Data Capture, Vanderbilt University, Nashville, TN, USA) [38]. Statistical analyses were performed using R version 4.2.2 statistical software. For the spatial analyses, the ArcGIS 10.7.1 geoprocessing program was used (Esri, Redlands, CA, USA) [39].

Entomological indices were calculated for the overall study area and for each community separately. The Ovitrap Positivity Index (OPI): percentage of ovitraps positive for eggs (number of recovered ovitraps with eggs / number of recovered ovitraps) was calculated separately for the intradomestic and peridomestic ovitraps. The Egg Density Index (EDI): average number of eggs per positive trap (total number of eggs from recovered ovitraps / number of recovered ovitraps) was also calculated.

Prior to statistical modelling, exploratory data analysis was performed to understand the distribution of continuous predictor variables and the outcome variable. Continuous variables were assessed for potential non-linear relationships with ovitrap egg counts, using univariable generalized additive models (GAMs) with a negative binomial distribution from the ‘mgcv’ package in R. Vegetation density showed an approximately linear relationship (effective degrees of freedom ≈1); therefore, it was retained as a continuous predictor. Monthly rainfall was included as a continuous variable to preserve variation across sampling periods and avoid loss of information from categorization. Additional methodological details are provided in S1 File. The outcome variable (*Ae. aegypti* egg count) was found to be overdispersed, and a negative binomial regression model was therefore used for analysis. Regression analyses were carried out for households with complete visual inspection and entomological data (n = 119).

Univariate analyses were carried out to individually examine the relationship between each predictor variable and the mosquito egg count outcome. The egg count data was used directly as opposed to converting it into a binary outcome to avoid a significant loss of information, given the high proportion of positive households. A negative binomial regression model was fitted for each predictor variable using the ‘glm.nb’ function from the ‘lme4’ package in R. Goodness-of-fit testing supported the negative binomial model as a better fit than a Poisson model.

A p-value below the conventional threshold of 0.05 was interpreted as indicating a statistically significant association between the predictor variable and mosquito egg abundance. However, any variables in the univariate analysis with a p-value below 0.25 were considered for the multivariate model (based on the Wald test for logistic regression which has a p-value cut-off point of 0.25).

Multivariate negative binomial generalised linear mixed-effects models (GLMMs) were fit using the ‘glmer.nb’ function from the ‘lme4’ package in R. Separate negative binomial GLMMs were initially developed for different categories of characteristics: sociodemographic, household characteristics, household practices and environmental characteristics (see Supplementary material). To account for temporal variation in environmental conditions during the ovitrap data collection process, monthly rainfall (mm) was included as a continuous covariate in the model.

Collinearity among predictor variables was assessed using variance inflation factors (VIF), and variables with high collinearity were adjusted for, or removed, as appropriate.

A random effect for household was included to enable the mosquito egg counts from both ovitraps (intradomicile and peridomicile) to be assessed individually, whilst accounting for within-household correlation, thus improving the accuracy of fixed-effect estimates and better reflecting the structure of the data. Additionally, the location of the ovitrap (intradomicile vs peridomicile) was included as a covariate to control for its potential influence on mosquito egg presence.

The ‘dredge’ function in the ‘MuMIn’ package in R was used for the selection process to obtain variables for the global multivariable model based on Akaike Information Criterion (AIC) ranking. For each category-specific model, the variables from the best-ranked model (i.e., the model with the lowest AIC) were selected for inclusion in the global model.

An interaction term between community and sewer proximity was evaluated as part of a sensitivity analysis to assess whether the effect of sewer proximity differed between study sites however, it was not retained in the final model (see Results). The model also included an offset for ovitrap exposure duration to account for differences in ovitrap deployment time (5, 6, or 7 days), which could influence egg count.

Spatial analysis was carried out in ArcGIS 10.7.1; the global Moran’s I statistic was used to test for spatial autocorrelation of egg counts due to the possible movement of female adult *Ae. aegypti* mosquitoes between households. The analysis was restricted to Nova Sussuarana, where ovitraps were installed within a three-week timeframe to minimize temporal variability. Marechal Rondon was excluded due to the extended ovitrap deployment period, which could introduce temporal variability. An inverse distance weighting approach was used to define spatial relationships, meaning that the spatial weights were defined such that closer households had a greater influence on one another. Neighbouring households were defined as those within a 30-metre radius of each other. This threshold was chosen based on the dispersion range of adult *Ae. aegypti* mosquitoes in urban areas and ensured that no households were isolated in the spatial weights matrix [26,41].

## Results

### Entomological indices

During the entomological survey, a total of 174 households were inspected across both communities with 38 households inspected in Nova Sussuarana and 136 households in Marechal Rondon. A total of 348 ovitraps (intradomicile and peridomicile) were installed in the overall study area, 76 ovitraps (intradomicile and peridomicile) were installed in Nova Sussuarana and 272 ovitraps (intradomicile and peridomicile) in Marechal Rondon.

Overall, 153 (87.9%) of the intradomicile ovitraps were recovered intact, and 150 (86.2%) of the peridomicile ovitraps were recovered intact across both communities. Of the recovered intradomicile ovitraps, 85 were positive for the presence of eggs (OPI: 55.6%), and of the recovered peridomicile ovitraps, 123 were positive for the presence of eggs (OPI: 82.0%) showing a higher ovitrap positivity index for peridomicile ovitraps. Overall, 11,211 eggs were counted on the pallets (EDI: 53.9 eggs), as shown in Table 1. In Nova Sussuarana, 57.9% of the intradomicile ovitrap and 97.3% of the peridomicile ovitraps were positive for eggs, while in Marechal Rondon, the ovitrap positivity rates were 54.8% and 77.0% respectively.

**Table 1:** Frequency of entomological indices for surveyed households in the overall study area and the individual communities, Nova Sussuarana and Marechal Rondon, Salvador, Brazil, April to August 2023.

	Overall study area	Nova Sussuarana	Marechal Rondon
Total number of inspected households	174	38	136
**Intradomicile**			
Number of positive recovered ovitraps (%)	85/153 (55.6)	22/38 (57.9)	63/115 (54.8)
**Peridomicile**			
Number of positive recovered ovitraps (%)	123/150 (82.0)	36/37 (97.3)	87/113 (77.0)
**Overall**			
Total number of eggs in the ovitrap pallets	11211	4678	6533
Egg Density Index, average	53.9	80.7	43.6

### Household and environmental data

The visual inspection survey was conducted in 119 (68.4%) of the 174 households where ovitraps had been installed across the two study communities due to safety-related access constraints. These 119 households formed the analytic sample for univariate and multivariate regression analyses. Examples of environmental vulnerabilities identified during the visual inspection, including containers containing stagnant water and poor waste management, are shown in S3 Fig. The survey could not be completed in the remaining households due to violence and unrest within the communities, which meant that the visual inspection surveys had to be suspended. Additionally, access to certain households was restricted either due to refusal to participate or inability to locate residents after at least three attempts.

The corresponding sociodemographic data from the standardised questionnaire completed by the head-of-the-household (HOH) for each of these households was extracted and analysed along with the data from the visual inspection survey, as shown in Table 2.

**Table 2:** Summary of sociodemographic, household and environmental characteristics in the overall study area and the individual communities, Nova Sussuarna and Marechal Rondon, Salvador, Brazil, April to August 2023.

	Overall study area		Nova Sussuarana		Marechal Rondon	
	(N = 119)		(N = 38)		(N = 81)	
**Sociodemographic characteristic**	**Median**	**IQR**	**Median**	**IQR**	**Median**	**IQR**
Number of inhabitants per household	3	2-4	3	2-4	3	2-4
Number of rooms per household	2	2-5	5	5-6	2	2-2
**Sociodemographic characteristic**	**n (%)**		**n (%)**		**n (%)**	
HOH monthly income above the National Minimum Wage	81 (69)		26 (68)		55 (70)	
HOH completed secondary education	65 (55)		21 (55)		44 (54)	
**Housing characteristic**						
Roof material						
Tile	23 (19)		3 (8)		20 (25)	
Concrete	35 (29)		22 (58)		13 (16)	
Eternit	61 (51)		13 (34)		48 (59)	
Has paved entry	112 (94)		32 (84)		80 (99)	
Has yard	71 (60)		15 (39)		56 (69)	
Has shaded yard	32 (45)		2 (13)		30 (54)	
Has shaded patio	39 (33)		21 (55)		18 (22)	
Has flood barriers (elevated floor or permanent wall)	41 (34)		7 (18)		34 (42)	
Has mosquito screens on windows	8 (7)		6 (16)		2 (2)	
**Public services and household practices**						
Deposit garbage in front of house	37 (31)		16 (42)		21 (26)	
Garbage accumulation within 10 m of house	58 (49)		13 (34)		45 (56)	
Garbage collections per week						
<3 times per week	6 (5)		1 (3)		5 (6)	
≥3 times per week	113 (95)		37 (97)		76 (94)	
Has water storage tank (peridomicile)	78 (66)		21 (55)		57 (70)	
Improper cover on water storage tank	10 (13)		4 (19)		6 (11)	
Open plastic containers	60 (50)		17 (45)		43 (53)	
Flowerpots	16 (13)		10 (26)		6 (7)	
Other containers	11 (9)		4 (11)		7 (9)	
Presence of standing water in open containers	37 (56)		16 (70)		21(49)	
**Environmental characteristic**						
Has vacant lot within 10 m	44 (37)		8 (21)		36 (44)	
Has open sewer within 10 m	62 (52)		34 (89)		28 (35)	
Has sewerage vulnerability within 30 m	33 (28)		26 (68)		7 (9)	
Has storm drain vulnerability within 30 m	59 (50)		30 (79)		29 (36)	

Of the 278 surveyed households, the majority (69%) of heads-of-households reported an income above the national minimum wage (approximately R$1200), and 55% had completed secondary education. Most households had paved entries (94%), and 60% had a yard, with nearly half (45%) of these yards being shaded. Water supply interruption within the past 30 days was reported by 47% of the HOHs. Water storage was common, with 75% of households storing water indoors and 66% having peri-domestic water storage tanks, 13% of which were improperly covered. Plastic containers were the most frequently observed open containers in peri-domestic environments, found at 50% of households.

Notable differences between the two study communities were observed: 89% of households in Nova Sussuarana were located within 10m of an open sewer, compared to 35% in Marechal Rondon. Similarly, storm drain vulnerability (within 30 m) of surveyed households was more prevalent in Nova Sussuarana (79%) than in Marechal Rondon (36%).

Summary statistics for the average mosquito egg count by each sociodemographic, household, and environmental characteristic are provided in S4 Table.

### Landcover Features

Supervised classification of a Worldview-3 satellite image revealed that impervious surfaces were the most common landcover category in both Nova Sussuarana and Marechal Rondon as shown in Fig 3, followed by vegetation. Within 30 m buffers around surveyed households, on average, the landcover was comprised of 62% (IQR: 54–68%) impervious surface cover and 28% (IQR: 20–35%) vegetation cover. The classification had a high accuracy (94.17%) and a Cohen’s Kappa score of 0.88, indicating a high level of agreement between the supervised landcover classification map and the reference data, reinforcing the reliability and accuracy of the classification results.

**Fig 3:**
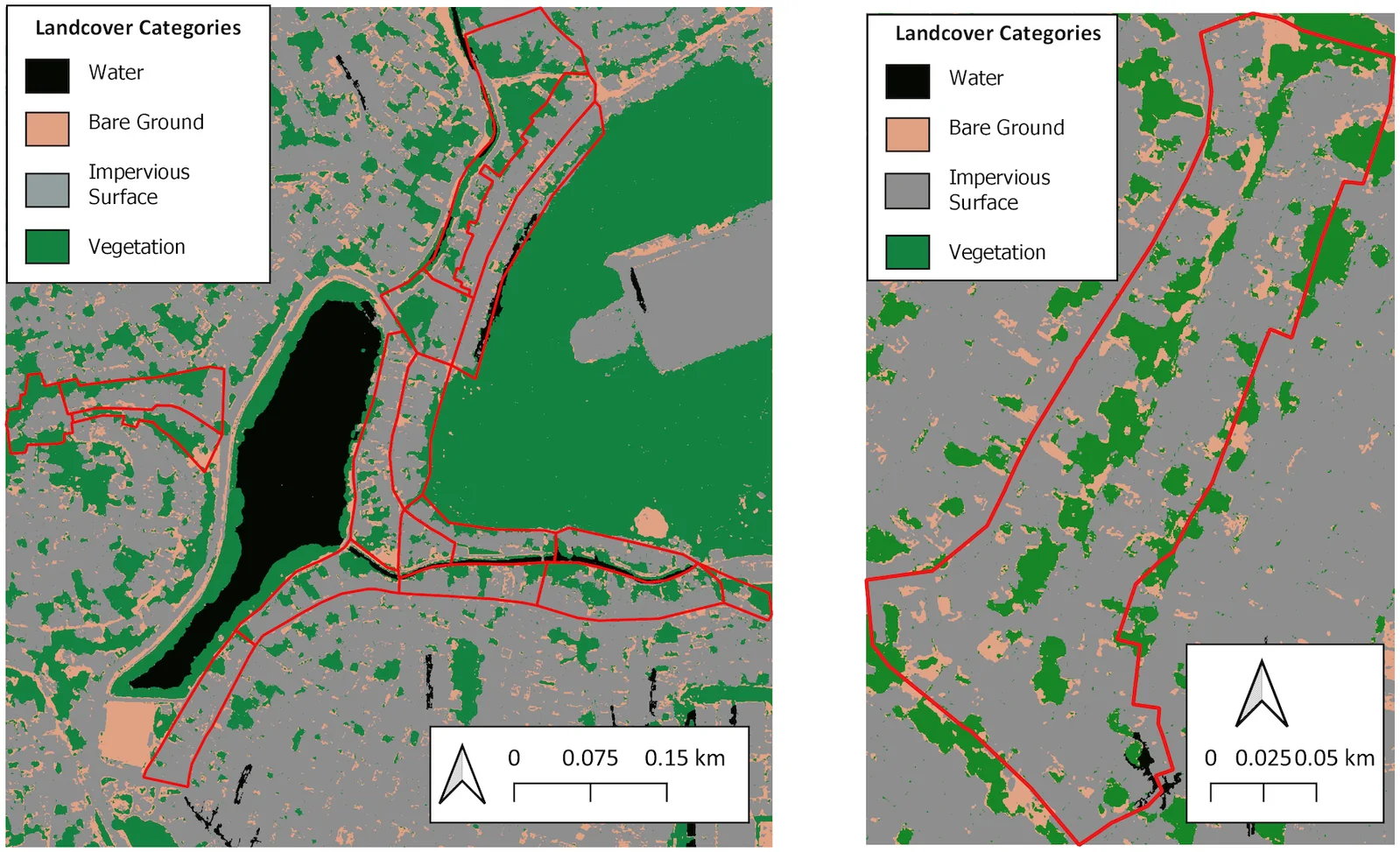
Landcover classification of the study sites (A) Marechal Rondon and (B) Nova Sussuarana derived from WorldView-3 satellite imagery (DigitalGlobe/Maxar Technologies. Used with permission. July 2023; 0.31 m panchromatic, 1.24 m multispectral resolution). The figure presents a derived raster landcover classification distinguishing water, bare ground, impervious surface, and vegetation.

### Univariate analysis results

Univariate negative binomial models were fitted to assess the association between individual socio-demographic, household, and environmental factors and mosquito egg abundance. The results of these analyses are shown in Table 3.

**Table 3:** Univariate negative binomial regression models for sociodemographic, household and environmental characteristics, and mosquito egg abundance.

Characteristic	IRR^a^	95% CI^a^	p-value
HOH monthly income above the National Minimum Wage	1.50	0.81-2.67	0.18
HOH completed secondary education	1.41	0.80-2.44	0.23
Number of inhabitants per household	0.93	0.77-1.14	0.41
Number of rooms per household	1.13	0.98-1.31	0.061
**Has Eternit roofing**	**0.51**	**0.29-0.88**	**0.016**
Has paved entry	0.50	0.12-1.36	0.24
Has yard	0.74	0.42-1.29	0.29
Has shaded yard	0.95	0.48-1.89	0.87
Has shaded patio	0.92	0.52-1.68	0.78
Has mosquito screens on windows	2.21	0.85-8.12	0.16
Observed mosquitoes in house	0.66	0.30-1.30	0.26
**Has flood barriers**	**0.46**	**0.26-0.84**	**0.009**
**Deposit garbage in front of house**	**2.91**	**1.65-5.35**	**<0.001**
Has garbage accumulation within 10 m of house	1.14	0.66-1.99	0.64
Garbage collection ≥3 times per week	0.71	0.16-2.04	0.59
Water supply is interrupted	0.73	0.42-1.27	0.26
Stores water (intradomicile)	1.15	0.59-2.10	0.66
Has water storage tank (peridomicile)	1.19	0.66-2.09	0.55
Has improper cover on water storage tank	0.53	0.26-1.18	0.10
**Has open plastic containers**	**1.52**	**0.87-2.64**	**0.14**
Has flowerpots	1.87	0.89-4.64	0.13
Has other open containers	1.45	0.64-4.08	0.42
Presence of standing water in open containers	1.92	0.97-3.77	0.058
Vacant lot within 10 m of house	0.90	0.51-1.65	0.72
**Open sewer within 10 m of house**	**1.94**	**1.12-3.35**	**0.017**
Sewerage vulnerability within 30 m of house	1.43	0.80-2.70	0.25
Storm drain vulnerability within 30 m of house	1.31	0.75-2.28	0.34
**Percentage of vegetation cover within 30 m of house**	**0.97**	**0.95-1.00**	**0.033**
**Total monthly precipitation (mm)**	**1.03**	**1.01-1.04**	**<0.001**
**Location of ovitrap (peridomicile)**	**2.60**	**1.51-4.47**	**<0.001**

^a^IRR = Incidence Rate Ratio, CI = Confidence Interval.

The head-of-the-household having a monthly income above the national minimum wage and the head-of-the-household completing secondary education were both positively associated with mosquito egg abundance (IRR = 1.50, 95%CI = 0.81-2.67, p = 0.18) (IRR = 1.41, 95%CI = 0.80-2.44, p = 0.23). The p-values were below 0.25, therefore, they were considered in the selection process for the multivariate model, as interactions with other variables could alter their significance.

Eternit roof material and flood barriers were negatively associated with mosquito egg abundance, suggesting that they have a protective effect on mosquito egg abundance (Eternit: IRR = 0.51, 95% CI = 0.29-0.88, p = 0.016; Flood barriers: IRR = 0.46, 95% CI = 0.26-0.84, p = 0.009). Households with window screens had a higher mosquito egg abundance (IRR = 2.21, 95% CI = 0.85-8.12, p = 0.16). The p-value was below 0.25, so it was included in further analysis.

Depositing garbage at the front of the house prior to garbage collection was positively associated with mosquito egg abundance, suggesting that it increases the risk of mosquito egg abundance (IRR = 2.91, 95% CI = 1.65-5.35, p < 0.001).

The presence of plastic open containers in the peri-domestic environment was positively associated with increased mosquito egg abundance (IRR = 1.52, 95% CI = 0.87-2.64, p = 0.14). The p-value was below 0.25; therefore, it was considered in the selection process for the multivariable model. Households with flowerpots in the peri-domestic environment had a 1.87 times higher incidence rate of mosquito egg abundance than those without flowerpots, and the p-value was below 0.25 (IRR = 1.87, 95%CI = 0.89- 4.64, p = 0.13). Living in close proximity (within 10 m) to an open sewer was positively associated with mosquito egg abundance and was statistically significant (IRR = 1.94, 95% CI = 1.12-3.35, p = 0.017).

Higher percentage of vegetation cover (within 30 m of the household) was negatively associated with mosquito egg abundance, which suggests that it is protective (IRR = 0.97, 95% CI = 0.95-1.00, p = 0.033). Despite the 95% CI including the null value of 1, the variable was retained in the multivariable analysis to further explore its potential association with mosquito egg abundance given the p-value <0.25.

Ovitraps located in the peridomicile environment were associated with significantly higher mosquito egg counts than intradomicile ovitraps (IRR = 2.60, 95%CI = 1.51-4.47, p < 0.001).

### Multivariate analysis results

In the socio-demographic model, no variables were found to be statistically significant. The household characteristics model retained the presence of flood barriers and roof material. The household practices model retained the presence of open plastic containers and flowerpots in the peri-domestic environment, and depositing garbage in front of the house prior to garbage collection. The environmental characteristics model retained living in close proximity to an open sewer (within 10 m). The VIF values for all predictors in each category-specific model were below 2, suggesting no multicollinearity issues.

The global model was constructed by including the significant predictors from the best-fitting model from each category-specific analysis, as determined by AIC. As a sensitivity analysis, an interaction term between sewer proximity and community was evaluated; however, its inclusion did not improve model fit (ΔAIC = 1.2), indicating equivalent empirical support relative to the simpler model. Therefore, the interaction term was excluded, and the more parsimonious model was retained. The final global model retained four predictors: ovitrap location, depositing garbage in front of the house, living in close proximity to an open sewer (within 10 m), the presence of open plastic containers in the peri-domestic environment, and monthly rainfall (mm). The model showed no multicollinearity (all VIFs < 1.1), and no overdispersion was detected (dispersion ratio = 0.373, p = 0.128), confirming the model assumptions were satisfied.

Results from the global multivariate negative binomial GLMM are shown in Table 4. The strongest predictors of *Ae. aegypti* egg abundance were ovitrap location and garbage accumulation. The IRR of mosquito egg abundance was 3.62 times as high for ovitraps located in the peridomicile environment compared to those located in the intradomicile environment (IRR = 3.62, 95%CI = 2.18-6.12, p = < 0.001). Depositing garbage in front of the house prior to garbage collection was also strongly associated with mosquito egg abundance (IRR = 3.35, 95%CI = 1.93-5.92, p = < 0.001).

**Table 4:** Results from the global multivariate negative binomial generalised linear mixed-effects model (GLMM) for characteristics associated with mosquito egg abundance.

Characteristic	IRR^a^	95%CI^a^	p-value
Open plastic containers	1.65	0.99-2.75	0.089
Deposit garbage in front of house	3.35	1.93-5.92	**<0.001**
Open sewer within 10 m of house	2.44	1.48-3.99	**0.001**
Monthly rainfall	1.57	1.21-1.98	**0.001**
Location of ovitrap (peridomicile)	3.62	2.18-6.12	**<0.001**

Living in close proximity to an open sewer (within 10 m) and monthly rainfall (mm) were also strong predictors of *Ae. aegypti* egg abundance. The incidence rate of *Ae. aegypti* egg abundance was 2.44 times as high for households that were in close proximity to an open sewer (within 10 m) than households that were further away (IRR = 2.44, 95%CI = 1.48-3.99, p = 0.001). Additionally, each unit increase in monthly rainfall was associated with 1.57 times higher *Ae. aegypti* egg abundance (IRR = 1.57, 95%CI = 1.21-1.98, p = 0.001).

The presence of open plastic containers in the peri-domestic environment was associated with higher incidence rate of *Ae. aegypti* egg abundance (IRR = 1.65, 95%CI = 0.99-2.75, p = 0.089), although it did not reach statistical significance, it improved the performance of the model. Further studies with a larger sample size are required to determine the effect of open plastic containers on mosquito egg abundance.

### Spatial analysis results

The global Moran’s I analysis did not show any significant spatial autocorrelation in ovitrap egg counts between the surveyed households in Nova Sussuarana (Moran’s I = 0.321, z-score = 1.84, p = 0.0665). The p-value was above the conventional 0.05 threshold, indicating no clear evidence of spatial clustering between neighbouring households.

## Discussion

This study highlights the burden of *Ae. aegypti* mosquito infestation in two disadvantaged communities of Salvador, Brazil. Key household and environmental factors associated with increased *Ae. aegypti* egg abundance included depositing garbage in front of the house, living in close proximity to an open sewer (within 10 m), monthly rainfall (mm), ovitrap location, and the presence of open plastic containers in the peri-domestic environment.

Ovitraps located in the peri-domestic environment had higher *Ae. aegypti* egg counts than those indoors, consistent with findings from Indonesia and South Texas [42,43]. Peri-domestic environments likely provide more favourable conditions for mosquito breeding due to a greater availability of artificial containers, which support “skip-oviposition” behaviour, as females can distribute their eggs across multiple sites. The inclusion of monthly rainfall in the model accounts for temporal variation across the study period, with higher rainfall likely increasing the availability of water-filled containers in the peri-domestic environments, consistent with the observed association between monthly rainfall (mm) and increased mosquito egg abundance. Longitudinal studies should be conducted to further investigate this relationship, as seasonal variation has been found to influence peri-domestic mosquito abundance [44].

The presence of open plastic containers in the peri-domestic environment was associated with increased *Ae. aegypti* egg abundance, which supports the role of artificial containers as key breeding sites for *Ae. aegypti*. These containers can collect and retain water from rainfall, creating ideal breeding sites for *Ae. aegypti* mosquitoes, which prefer to lay their eggs in stagnant water. Previous studies have identified discarded containers in the peri-domestic environment as a risk factor for *Ae. aegypti* immature abundance and persistence [21,41,45]. In Salvador, Souza et al. [21] found that buckets and tanks were the most common types of water-holding containers, and the most productive for *Ae. aegypti* immature growth, in the private area of households in urban environments. These results highlight the need for environmental manipulation strategies, such as removing or covering open containers, to reduce available vector breeding sites.

Depositing garbage in front of households was also strongly associated with *Ae. aegypti* egg abundance, aligning with previous studies linking poor waste management and mosquito infestation [46,47]. Garbage can accumulate rainwater, thereby serving as ideal oviposition sites. This improper waste storage suggests that these communities lack proper infrastructure and waste management, such as sufficient garbage bins or other receptacles for storing waste. Although 95% of the head-of-the-households (HOHs) reported that garbage collection occurred more than three times per week, the service may potentially be insufficient leading to waste left behind or certain areas not adequately covered. Addressing gaps through improved collection services and the provision of household receptacles is crucial for vector control efforts.

Proximity to an open sewer (within 10 m) was strongly associated with *Ae. aegypti* egg abundance. The environment surrounding open sewers may contain sources of stagnant water, providing ideal breeding grounds for *Ae. aegypti,* as this species has been found to exploit both clean and polluted water sources for breeding [48,49]. Previous studies have linked the presence of open sewage to increased dengue incidence in Brazil [50]. However, the role of open sewers as a direct mosquito breeding site remains uncertain. Instead, open sewers may influence mosquito egg counts in surrounding households through alternative indirect mechanisms. For example, during periods of heavy rainfall, open sewers may overflow, filling discarded open containers in the surrounding area and creating microhabitats for mosquito breeding. Alternatively, female *Ae. aegypti* mosquitoes may preferentially oviposit in cleaner water sources in and around households to avoid the polluted sewage water. These findings highlight the importance of improving sanitation infrastructure in addition to promoting community awareness and participation in sanitation efforts.

Spatial analysis revealed no significant clustering of *Ae. aegypti* eggs in Nova Sussuarana, which suggests that *Ae. aegypti* populations remain near breeding sites and are influenced by local factors. This emphasises the importance of identifying and eliminating risk factors at the household level. Other studies have also found that *Ae. Aegypti* is highly localised in nature within urban environments [26,27]. However, the relatively small study area may have reduced the ability to detect spatial clustering. Additionally, the spatial patterns could not be detected in Marechal Rondon due to the temporal variation in ovitrap deployment. Future studies with larger samples could improve the robustness of the spatial analysis and provide better insights into the spatial pattern of *Ae. aegypti* in these study areas.

This study has several other limitations. The cross-sectional design prevented the analysis of temporal trends and seasonal effects on *Ae. aegypti* distribution. Furthermore, fieldwork was occasionally interrupted by flooding, community unrest, and restricted household access, limiting sample size and geographic scope. This introduced temporal variation in the data collection process, as the visual inspection survey had to be postponed ensuring the safety of the research team. Despite these challenges, efforts were made to minimise the impact of these delays by coordinating the timing of the visual inspection surveys with the collection of entomological data at each of the surveyed households. Additionally, monthly accumulated rainfall (mm) corresponding to each ovitrap sampling period was included as a covariate in the multivariate model. This aimed to reduce the potential confounding from temporal differences in data collection across the communities. Another limitation was that a control area without open sewers was not included; therefore, the influence of this risk factor could not be isolated from others.

There were a few limitations to the entomological data collection process. Ovitrap placement was limited to households, potentially missing public breeding sites. Some traps were lost or damaged, resulting in missing data. Additionally, the Egg Density Index does not account for adult mosquito abundance or the actual risk of arbovirus transmission. There was minimal potential for misclassification of eggs; however, based on prior studies and preliminary observations, almost all eggs were likely *Aedes aegypti*.

## Conclusions

Overall, this study highlights the critical role of environmental sanitation characteristics in facilitating *Aedes aegypti* breeding. Specifically, the presence of open plastic containers, accumulated garbage, and proximity to open sewers were identified as key risk factors. Targeted interventions addressing these conditions could be instrumental in reducing mosquito populations and, in turn, the risk of arboviral disease transmission in vulnerable communities.

Environmental sanitation interventions have already proven effective in Salvador, such as storm drain interventions that prevent water accumulation and debris buildup, thereby reducing the amount of stagnant water and the proportion of storm drains containing *Ae. aegypti* immatures [21].

An integrated approach that combines biological interventions, such as *Wolbachia-*infected mosquitoes, with targeted environmental management could increase the sustainability of mosquito control measures. This aligns with WHO recommendations, which advocate for an integrated vector management approach as opposed to single interventions to improve efficacy and ensure ecological balance [25]. This is increasingly more important in the context of climate change, and the rapid expansion of arboviral diseases, as disadvantaged communities require sustainable and resilient vector control strategies to address these challenges.

## Supporting information

S1 Table Timeline of data collection activities. (DOCX)

S1 Fig Socio-demographic and household practices questionnaire. Questions selected from the survey questionnaire administered by the ‘Sanitation Interventions in Prevention of Urban Leptospirosis’ project to gather information on socio-demographics and household practices during the household census that could be associated with *Ae. aegypti* breeding within households.
(DOCX)

S2 Table Socio-demographic data utilised in this study. The data extracted from the socio-demographic survey that were included in the analysis.
(DOCX)

S2 Fig Visual inspection survey for environmental risk factors. The visual inspection survey used during data collection to identify potential environmental risk factors for *Ae. aegypti* mosquitoes in the peri-domestic environment (within a 10 m radius of each household).
(DOCX)

S3 Table Household survey data utilised in this study. The data extracted from the visual inspection survey that were included in the analysis.
(DOCX)

S1 File Exploratory data analysis prior to statistical modelling. Exploratory data analysis examining the distribution of continuous predictor variables and the outcome variable.
(DOCX)

S3 Fig Photographs of environmental risk factors in the communities. Photographs of containers with stagnant water that could act as *Ae. aegypti* breeding sites in the peri-domestic environment of surveyed households.
(DOCX)

S4 Table Average mosquito egg counts by sociodemographic, household and environmental characteristics. Summary statistics for average mosquito egg counts in relation to sociodemographic, household, and environmental characteristics in the study areas.
(DOCX)

S2 File The R script for the univariate and multivariate models. (DOCX)
