# Supplementary material for: Household and community drivers of Aedes aegypti infestation in tropical cities: Guiding community-led interventions
Source: PLoS Negl Trop Dis. 2026 Oct 1;20(10):e0014728. doi: 10.1371/journal.pntd.0014728 (PMC13638034; doi:10.1371/journal.pntd.0014728)
Supplement: Supplementary file 1 [file pntd.0014728.s001.docx]

A timeline summarising when each dataset was collected across the two communities.

Timeline for data collection in Marechal Rondon:

| Study Phase: | Activity: | Start Date: | End Date: |
| --- | --- | --- | --- |
| Fieldwork | Community-level environmental engineering data collection | October 2022 | October 2022 |
| Fieldwork | Socio-demographic data collection | February 2023 | June 2023 |
| Fieldwork | Ovitrap installation | April 2023 | December 2023 |
| Fieldwork | Household-level environmental visual inspection survey | April2023 | December 2023 |

Timeline for data collection in Nova Sussuarana:

| Study Phase: | Activity: | Start Date: | End Date: |
| --- | --- | --- | --- |
| Fieldwork | Community-level environmental engineering data collection | May 2023 | May 2023 |
| Fieldwork | Socio-demographic data collection | October 2021 | October 2021 |
| Fieldwork | Ovitrap installation | March 2023 | April 2023 |
| Fieldwork | Household-level environmental visual inspection survey | March 2023 | April 2023 |
