# Supplementary material for: Household and community drivers of Aedes aegypti infestation in tropical cities: Guiding community-led interventions
Source: PLoS Negl Trop Dis. 2026 Oct 1;20(10):e0014728. doi: 10.1371/journal.pntd.0014728 (PMC13638034; doi:10.1371/journal.pntd.0014728)
Supplement: Supplementary file 2 [file pntd.0014728.s002.docx]

**Socio-demographic and household practices questionnaire.** Questions selected from the survey questionnaire administered by the ‘Sanitation Interventions in Prevention of Urban Leptospirosis’ project to gather information on socio-demographics and household practices during the household census that could be associated with *Ae. aegypti* breeding within households.
