# Supplementary material for: Household and community drivers of Aedes aegypti infestation in tropical cities: Guiding community-led interventions
Source: PLoS Negl Trop Dis. 2026 Oct 1;20(10):e0014728. doi: 10.1371/journal.pntd.0014728 (PMC13638034; doi:10.1371/journal.pntd.0014728)
Supplement: Supplementary file 3 [file pntd.0014728.s003.docx]

**Socio-demographic data utilised in this study.** The data extracted from the socio-demographic survey that were included in the analysis.

**S2- The socio-demographic data utilised in this study included:**

| Characteristic: | Response: |
| --- | --- |
| Number of inhabitants per household | Number of adults and children |
| Number of rooms per household | Number of rooms |
| Paved access to household | Yes/No |
| Head-of-the-household income | Enter income |
| Head-of-the-household level of education (last school year passed) | Enter last school year completed |

Other data relating to household practices was also extracted and used in this study including:

| Characteristic: | Response: |
| --- | --- |
| Frequency of garbage collections per week | Once, twice, three or more times |
