# Supplementary material for: Household and community drivers of Aedes aegypti infestation in tropical cities: Guiding community-led interventions
Source: PLoS Negl Trop Dis. 2026 Oct 1;20(10):e0014728. doi: 10.1371/journal.pntd.0014728 (PMC13638034; doi:10.1371/journal.pntd.0014728)
Supplement: Supplementary file 4 [file pntd.0014728.s004.docx]

**Visual inspection survey for environmental risk factors.** The visual inspection survey used during data collection to identify potential environmental risk factors for *Ae. aegypti* mosquitoes in the peri-domestic environment (within a 10 m radius of each household).
