# Supplementary material for: Household and community drivers of Aedes aegypti infestation in tropical cities: Guiding community-led interventions
Source: PLoS Negl Trop Dis. 2026 Oct 1;20(10):e0014728. doi: 10.1371/journal.pntd.0014728 (PMC13638034; doi:10.1371/journal.pntd.0014728)
Supplement: Supplementary file 5 [file pntd.0014728.s005.docx]

The data extracted from the visual inspection survey that were included in the analysis.

(i) identification of household characteristics

| Characteristic: | Response: |
| --- | --- |
| Location of house | Slope, hilltop, valley |
| Floor material | Tile/stone, parquet flooring, cement, reclaimed wood, clay/earth, other |
| Roof material | Roof tile, concrete slab, Eternit, other |
| Presence of a shaded patio | Yes/No |
| Presence of a yard | Yes/No |
| Whether the yard was shaded | Yes/No |
| Presence of mosquito window screens | Yes/No |
| Presence of flood barriers at household | Yes/No |
| Type of flood barriers present | Permanent wall, temporary plastic barriers, temporary wooden barriers, temporary cement barriers, elevated floor, other |

(ii) characterisation of water storing practices

| Characteristic: | Response: |
| --- | --- |
| Presence of an enclosed tank for water storage | Yes/No |
| Presence of a proper lid on water storage tank | Yes/No |

(iii) identification of potential mosquito breeding sites preferentially for *Ae. aegypti* in the peridomestic environment of households

| Characteristic: | Response: |
| --- | --- |
| Deposit garbage in front of house | Yes/No |
| Presence of open containers outside the house | Yes/No |
| Type of open containers present | Plastic containers, discarded tires, PVC tanks, flowerpots, other |
| Presence of standing water in open container | Yes/No |

(iv) identification of environmental characteristics in the peri-domestic environment (within 10 m) of households

| Characteristic: | Response: |
| --- | --- |
| Presence of a vacant lot | Yes/No |
| Presence of an open sewer | Yes/No |
| Presence of storm drains | Yes/No |
| Whether the storm drain was open | Yes/No |
| Whether the storm drain was blocked | Yes/No |
| Whether the storm drain was damaged | Yes/No |
