# Supplementary material for: Household and community drivers of Aedes aegypti infestation in tropical cities: Guiding community-led interventions
Source: PLoS Negl Trop Dis. 2026 Oct 1;20(10):e0014728. doi: 10.1371/journal.pntd.0014728 (PMC13638034; doi:10.1371/journal.pntd.0014728)
Supplement: Supplementary file 6 [file pntd.0014728.s006.docx]

**Exploratory data analysis prior to statistical modelling.** Exploratory data analysis examining the distribution of continuous predictor variables and the outcome variable.

Prior to statistical modelling, exploratory data analysis was performed to understand the distribution of continuous predictor variables and the outcome variable. A histogram and quantile-quantile (Q-Q) plot were generated to assess the distribution of the proportion of vegetation cover (within 30 m) around households to determine whether transformation was required before further analysis.

**Distribution of Vegetation Cover (%)**

10

20

30

40

50

0

5

1

0

1

5

2

0

Percentage of Vegetation Cover (%)

A histogram showing the distribution of the percentage of vegetation cover within 30m of households

**Q−Q Plot of Vegetation Cover (%)**

−2

−1

0

1

2

1

0

2

0

3

0

4

0

5

0

Theoretical Quantiles

A Q-Q plot showing the distribution of the percentage of vegetation cover within 30m of households. The observed data points are plotted against the expected values from a theoretical normal distribution

The Q-Q plot adhered to a straight line indicating approximate normality, so no transformation was necessary.

The proportion of vegetation cover (within 30 m) was also assessed for potential non-linear relationships with ovitrap egg counts, using univariable generalised additive models (GAMs) with a negative binomial distribution from the ‘mgcv’ package in R. A quadratic term and GAM smooth were tested to check for non-linearity. Both tests indicated an approximately linear association (effective degrees of freedom ≈ 1, likelihood ratio test p = 0.70), so proportion of vegetation cover (within 30 m) was retained as a continuous predictor in the multivariable analysis.

The distribution of the outcome variable was also investigated. The histogram for the mosquito egg counts demonstrated that the data was widely spread-out which suggested overdispersion.

A histogram showing the distribution of mosquito egg counts. A large proportion of the counts are concentrated near the zero, indicating a skewed distribution with potential overdispersion in the data.

The DHARMa nonparametric test compared the dispersion of simulated residuals to the observed residuals. The output for a Poisson regression model, which is usually used to model count data, showed that this distribution family was not a good fit for the data as shown below.

**DHARMa nonparametric dispersion test via sd of**

**residuals fitted vs. sim**

**ulated**

F

r

e

q

u

e

n

c

y

0

5

10

15

0

5

1

0

1

5

2

0

2

5

Simulated values, red line = fitted model. p−value (two.sided) = 0

The output of the DHARMa nonparametric dispersion test for a Poisson regression model

The dispersion was 159.93 and the p-value was < 2.2e-16 which suggests that the dispersion of the residuals was significantly different from what would be expected under the null hypothesis of a correctly specified model. This provided evidence of overdispersion in the model residuals, the variability in the outcome variable (mosquito egg count) was greater than would be expected under a Poisson distribution, which suggested that it was not an appropriate model for this data.

The output of the DHARMa nonparametric test for a negative binomial regression model showed that this distribution family was a much better fit for the data as shown below.

Negative binomial regression accommodates for the excess variability in the data.

**Figure 10-** The output of the DHARMa nonparametric dispersion test for a negative binomial model

The dispersion was 0.856 and the p-value was 0.848 which suggests that the dispersion of the residuals is not significantly different from what would be expected under the null hypothesis of a correctly specified model. This suggests that the negative binomial regression model adequately captures the variability in the response variable and fits the data well.
