# Supplementary material for: Household and community drivers of Aedes aegypti infestation in tropical cities: Guiding community-led interventions
Source: PLoS Negl Trop Dis. 2026 Oct 1;20(10):e0014728. doi: 10.1371/journal.pntd.0014728 (PMC13638034; doi:10.1371/journal.pntd.0014728)
Supplement: Supplementary file 7 [file pntd.0014728.s007.docx]

| **A)** | **B)** |
| --- | --- |
| **C)** | **D)** |

**Photographs of environmental risk factors in the communities.** Photographs of containers with stagnant water that could act as *Ae. aegypti* breeding sites in the peri-domestic environment of surveyed households.

A) A water storage container, with an improperly closed lid, typical in the households within the study communities
B) An open water storage container in the yard of a surveyed household
C) Mosquito immatures present in the standing water of the open storage container
D) Discarded open plastic containers holding stagnant water in the peri-domestic environment of surveyed households
