# Supplementary material for: Household and community drivers of Aedes aegypti infestation in tropical cities: Guiding community-led interventions
Source: PLoS Negl Trop Dis. 2026 Oct 1;20(10):e0014728. doi: 10.1371/journal.pntd.0014728 (PMC13638034; doi:10.1371/journal.pntd.0014728)
Supplement: Supplementary file 8 [file pntd.0014728.s008.docx]

**Average mosquito egg counts by sociodemographic, household and environmental characteristics.** Summary statistics for average mosquito egg counts in relation to sociodemographic, household, and environmental characteristics in the study areas.

| **Sociodemographic characteristic** | **Mosquito egg count** Mean (SD) | | **Mosquito egg count** Median (IQR) |
| --- | --- | --- | --- |
| HOH monthly income above  National Minimum Wage  Yes  No | 44.6 (71.6)  28.9 (28) | | 19 (4-44) 20 (8-44) |
| HOH completed secondary  education  Yes  No | 45.2 (65.5)  32.5 (55.7) | | 22 (5.5-54.5)  17.5 (3-33) |
| **Housing characteristic** | | |  |
| Roof material  Tile  Concrete  Eternit | 62 (97.9)  48.2 (67.2)  28 (35.2) | | 19 (6-40)  32 (8-52.5)  17 (3-41) |
| Paved entry Yes  No | 36.8 (58.7)  81.8 (88.7) | | 19 (4-42.5)  43 (26-128) |
| Yard  Yes  No | 34.9 (58.6)  46.7 (65.5) | | 19.5 (5.8-34.8)  17 (3-57) |
| Shaded yard Yes  No | 33.2 (62.5)  36.2 (56.3) | | 20 (4-27)  19 (6-43.5) |
| Shaded patio  Yes  No | 38.4 (52.3)  40.2 (66.1) | | 23 (5.5-38)  19 (4-43) |
| Flood barriers  Yes  No | 24.1 (28.7)  46.9 (70.8 | | 12 (1.8-41.5)  22 (6-48) |
| Mosquito screens on windows  Yes  No | 85.8 (109)  36.1 (55.6) | | 38 (12.2-121) 19 (4-43) |
| Observed mosquitoes in house  Yes  No |  | 36.8 (58.6)  52.6 (73.5) | 18 (4-40)  31 (9-53.2) |
| **Public services and household practices** | |  |  |
| Deposit garbage in front of house  Yes  No |  | 71.3 (85.5) 25.4 (40) | 43 (12-81)  16 (3.2-32) |
| Garbage accumulation within 10 m of house  Yes  No |  | 44 (62.4)  35.4 (60.8) | 23 (7.5-46)  16.5 (3-42.5) |
| Garbage collection  <3 times per week  ≥3 times per week |  | 52.5 (82.3)  39.1 (60.5) | 20 (4.8-49.5)  19 (4-43) |
| Water supply is interrupted  Yes  No |  | 33.6 (59.4)  45.1 (63.2) | 12 (2-36)  22 (9.5-53.5) |
| Stores water (intradomicile)  Yes  No |  | 41.6 (63.6)  34.5 (56.4) | 17 (4-46)  24 (2-43) |
| Water storage tank (peridomicile)  Yes  No |  | 40.4 (61.7)  38.2 (61.7) | 23 (4-43)  19 (4-47.2) |
| Improper cover on water storage tank  Yes  No |  | 27.3 (17.6)  42.8 (65.9) | 17.5 (3.2-48.5)  20.5 (18-38.8) |
| Open plastic containers  Yes  No |  | 52.3 (76.2)  26.2 (36.5) | 24 (8-54.5)  16 (2.5-33) |
| Flowerpots Yes  No |  | 68.7 (90.3) 35.2 (55) | 32 (13.5-71)  19 (4-42.5) |
| Other open containers  Yes  No |  | 71.1 (98.5)  36.5 (56.4) | 33.5 (21.2-52.2)  18 (4-43.5) |
| Standing water in open containers  Yes  No |  | 59.4 (83.2)  42.7 (64.3) | 24.5 (8.8-55.8)  20.5 (7.8-45.8) |
| **Environmental characteristic** | |  |  |
| Vacant lot within 10 m of house  Yes  No | 35.9 (47.9)  41.5 (67.5) | | 20 (5.5-50.8)  19 (3-42) |
| Open sewer within 10 m of house  Yes  No | 50.9 (69.8)  27.3 (48.5) | | 24 (6-58.5)  16.5 (3.2-31.5) |
| Sewerage vulnerability within 30 m of house  Yes  No | 50.8 (69.5)  35 (57.6) | | 23 (6-57)  18.5 (3-37) |
| Storm drain vulnerability within  30 m of house  Yes  No | 44.9 (68.5)  34.6 (54.1) | | 19 (4.5-51)   19.5 (4-41) |

The data was positively skewed and the large standard deviations and interquartile ranges (IQRs) further demonstrated substantial variability within the data. The mean mosquito egg count was higher for households with a head-of-the-household (HOH) that had a monthly income above the National Minimum Wage (NMW), but the median was lower compared to households with incomes below the threshold; the skewness indicates outliers among higher-income households.

Higher mosquito egg counts were observed in households with unpaved entries, tile or concrete roofing, and no flood barriers, suggesting that these features might be linked to environmental conditions that promote *Ae. Aegypti* breeding. Unexpectedly, mosquito egg counts were also higher in households with window screens, which suggests that they may be improperly maintained or ineffective.

Households that deposited garbage at the front of the house prior to collection, and those with open plastic containers, flowerpots, or other open containers, all had higher mosquito egg counts.

The mean and median mosquito egg counts were higher for households within 10m of an open sewer, suggesting that this environmental characteristic could favour *Ae. Aegypti* breeding.
