# Supplementary material for: Household and community drivers of Aedes aegypti infestation in tropical cities: Guiding community-led interventions
Source: PLoS Negl Trop Dis. 2026 Oct 1;20(10):e0014728. doi: 10.1371/journal.pntd.0014728 (PMC13638034; doi:10.1371/journal.pntd.0014728)
Supplement: Supplementary file 9 [file pntd.0014728.s009.docx]

**The R script for the univariate and multivariate models.**

Mosquitoes <- read.csv("mosquitoes_final_ov.csv")

View(Mosquitoes)

summary(Mosquitoes)

Mosquitoes$community<-as.factor(Mosquitoes$community)

Mosquitoes$id_casa<-as.factor(Mosquitoes$id_casa)

Mosquitoes$guttering_dam<-as.factor(Mosquitoes$guttering_dam)

Mosquitoes$col_rainwater<-as.factor(Mosquitoes$col_rainwater)

Mosquitoes$rain_month<-as.numeric(Mosquitoes$rain_month)

Mosquitoes$rain_month_c <- scale(Mosquitoes$rain_month, center = TRUE, scale = TRUE)

Mosquitoes$days_ovi <- as.numeric(Mosquitoes$days_ovi)

Mosquitoes$storage_cover[Mosquitoes$storage_cover == 9] <- NA

\# Recode 0s as 1s, 1s as 0s in storage_cover

Mosquitoes$storage_cover <- ifelse(Mosquitoes$storage_cover == 0, 1,

ifelse(Mosquitoes$storage_cover == 1, 0,

Mosquitoes$storage_cover))

Mosquitoes$storage_cover<-as.factor(Mosquitoes$storage_cover)

Mosquitoes$open_container<-as.factor(Mosquitoes$open_container)

Mosquitoes$pvc_tanks<-as.factor(Mosquitoes$pvc_tanks)

Mosquitoes$flowerpots<-as.factor(Mosquitoes$flowerpots)

Mosquitoes$plastic_containers<-as.factor(Mosquitoes$plastic_containers)

Mosquitoes$other_container<-as.factor(Mosquitoes$other_container)

Mosquitoes$standing_water[Mosquitoes$standing_water == 9] <- NA

Mosquitoes$standing_water<-as.factor(Mosquitoes$standing_water)

Mosquitoes$drains_10m<-as.factor(Mosquitoes$drains_10m)

Mosquitoes$shaded_patio<-as.factor(Mosquitoes$shaded_patio)

Mosquitoes$water_stop<-as.factor(Mosquitoes$water_stop)

Mosquitoes$yard<-as.factor(Mosquitoes$yard)

\# Recode 9 to NA for shaded_yard

Mosquitoes$shaded_yard[Mosquitoes$shaded_yard == 9] <- NA

Mosquitoes$shaded_yard<-as.factor(Mosquitoes$shaded_yard)

Mosquitoes$vegetation_10m<-as.factor(Mosquitoes$vegetation_10m)

Mosquitoes$vacant_lot<-as.factor(Mosquitoes$vacant_lot)

Mosquitoes$window_screens<-as.factor(Mosquitoes$window_screens)

Mosquitoes$flood_barriers<-as.factor(Mosquitoes$flood_barriers)

Mosquitoes$vegetation_10m<-as.factor(Mosquitoes$vegetation_10m)

Mosquitoes$tp_barrier<-as.factor(Mosquitoes$tp_barrier)

Mosquitoes$material_floor<-as.factor(Mosquitoes$material_floor)

Mosquitoes$garbage_accum<-as.factor(Mosquitoes$garbage_accum)

Mosquitoes$sewer_close<-as.factor(Mosquitoes$sewer_close)

Mosquitoes$material_roof<-as.factor(Mosquitoes$material_roof)

Mosquitoes$material_roof_ed <- ifelse(Mosquitoes$material_roof == 3, 1, 0)

Mosquitoes$material_roof_ed<-as.factor(Mosquitoes$material_roof_ed)

Mosquitoes$pavement<-as.factor(Mosquitoes$pavement)

Mosquitoes$secondary_education<-as.factor(Mosquitoes$secondary_education)

Mosquitoes$mosquito<-as.factor(Mosquitoes$mosquito)

Mosquitoes$sewer_close<-as.factor(Mosquitoes$sewer_close)

Mosquitoes$flood_house<-as.factor(Mosquitoes$flood_house)

Mosquitoes$sewer_overflow<-as.factor(Mosquitoes$sewer_overflow)

Mosquitoes$garbage_house<- as.factor(Mosquitoes$garbage_house)

Mosquitoes$location_ov<- as.factor(Mosquitoes$location_ov)

Mosquitoes$imat<- as.factor(Mosquitoes$imat)

Mosquitoes$oviposit<- as.factor(Mosquitoes$oviposit)

\# Create a new binary variable indicating whether the household is within 30 meters of a sewerage vulnerability

Mosquitoes$sewer_vuln_30 <- ifelse(Mosquitoes$sewer_dist <= 30, 1, 0)

\# Convert the binary variable to a factor for categorical representation (optional)

Mosquitoes$sewer_vuln_30 <- as.factor(Mosquitoes$sewer_vuln_30)

\# Create a new binary variable indicating whether the household is within 30 meters of a storm drain vulnerability

Mosquitoes$storm_vuln_30 <- ifelse(Mosquitoes$storm_dist <= 30, 1, 0)

\# Convert the binary variable to a factor for categorical representation

Mosquitoes$storm_vuln_30 <- as.factor(Mosquitoes$storm_vuln_30)

\# Create the new binary variable 'garbage_front' from garbage_house

Mosquitoes$garbage_front <- ifelse(Mosquitoes$garbage_house == 1, 1, 0)

\# Convert the variable to a factor

Mosquitoes$garbage_front <- as.factor(Mosquitoes$garbage_front)

\# Assuming "9" represents missing data in the variable "collect_garbage"

Mosquitoes$collect_garbage[Mosquitoes$collect_garbage == 9] <- NA

Mosquitoes$collect_garbage <- as.factor(Mosquitoes$collect_garbage)

\# Convert 'collect_garbage' to numeric

Mosquitoes$collect_garbage <- as.numeric(as.character(Mosquitoes$collect_garbage))

\# Create a new binary variable based on the original categories where 1 represents 3 or more times per week and 0 represents less than 3 times per week

Mosquitoes$garbage_collections <- ifelse(Mosquitoes$collect_garbage >= 3, 1, 0)

\# Convert the variable to a factor

Mosquitoes$garbage_collections <- as.factor(Mosquitoes$garbage_collections)

\# Create a new binary variable 'income_nmw' where 1= above or equal to national minimum wage R$1200 and 0= below national minimum wage

Mosquitoes$income_nmw <- ifelse(Mosquitoes$household_income >= 1200, 1, 0)

\# Optional: Convert the binary variable to a factor

Mosquitoes$income_nmw <- as.factor(Mosquitoes$income_nmw)

\# Assuming 'inhabitants' and 'rooms' are variables in your dataset

\# Create a new variable for inhabitants per room

Mosquitoes$household_crowding <- Mosquitoes$inhabitants / Mosquitoes$rooms

\# Optionally, you can round the values to a certain number of decimal places

Mosquitoes$household_crowding <- round(Mosquitoes$household_crowding, 2)

Mosquitoes$log_crowding <- log(Mosquitoes$household_crowding)

Mosquitoes$veg_cat <- cut(Mosquitoes$proportion_veg_30,

breaks = c(0, 25, 50, 100),

include.lowest = TRUE,

labels = c("low", "medium", "high"))

table(Mosquitoes$veg_cat)

\# Check the first few rows of the dataset to verify the new variables

head(Mosquitoes)

variable_labels <- c(

income_nmw = "HOH income above National Minimum Wage",

secondary_education = "HOH completed secondary education",

inhabitants = "Number of inhabitants in household",

rooms = "Number of rooms in household",

material_roof_ed = "Roof material",

pavement = "Paved access to household",

yard = "Yard",

shaded_yard = "Shaded Yard",

shaded_patio = "Shaded patio",

window_screens = "Presence of mosquito screens on the windows",

mosquito = "Observed mosquitoes in the household",

flood_barriers = "Flood barriers",

collect_garbage = "Frequency of garbage collections per week",

garbage_collections = "3 or more",

garbage_front = "Deposit garbage in front of the house",

garbage_accum = "Garbage accumulation within 10m of the household",

col_rainwater = "Water storage tank (peridomicile)",

storage_cover = "Improper cover on water storage tank",

plastic_containers = "Plastic containers in the peridomestic environment",

flowerpots = "Flowerpots in the peridomestic environment",

other_container = "Other containers in the peridomestic environment",

standing_water = "Presence of standing water in open containers",

vacant_lot = "Vacant lot within 10m of the household",

sewer_close = "Open sewer within 10m of the household",

sewer_vuln_30 = "Sewerage vulnerability within 30m of the household",

storm_vuln_30 = "Storm drain vulnerability within 30m of the household",

proportion_veg_30 = "Percentage of vegetation cover",

veg_cat = "Percentage of vegetation cover (high, medium, low)",

rain_month = "Total monthly precipitation",

location_ov = "Location of the ovitrap (intradomicile/peridomicile)"

)

View(Mosquitoes)

library(gtsummary)

library(flextable)

library(dplyr)

library(MASS)

library(lme4)

library(ggplot2)

options(na.action = "na.omit") ####This means that whenever R encounters missing values in functions that perform analyses on data frames, it will omit any rows containing 'NA' values from the analysis

summary_result <- summary(Mosquitoes)

write.csv(summary_result, file = "summary_output_average.csv")

\### unifactorial models for mosquito abundance by individual factors

uni <- Mosquitoes %>%

dplyr::select(income_nmw, secondary_education, inhabitants, rooms, material_roof_ed,

pavement, yard,	shaded_yard, shaded_patio, window_screens, mosquito,

flood_barriers, garbage_front, garbage_accum, garbage_collections, water_interruption, storewater, col_rainwater,	storage_cover,

plastic_containers,	flowerpots, other_container, standing_water,

vacant_lot, sewer_close, sewer_vuln_30, storm_vuln_30,

proportion_veg_30, veg_cat, rain_month, location_ov, ov_eggs ) %>%

tbl_uvregression(

method = MASS::glm.nb, ###### This is a glm for negative binomial distribution

y = ov_eggs,

formula = "{y} ~ {x}",

method.args = list(),

exponentiate = TRUE,

pvalue_fun = ~style_pvalue(.x, digits = 2),

label = variable_labels

) %>%

bold_p()%>%

as_flex_table() %>%

flextable::save_as_docx(., path = "univariable_ovitraps_PLOS.docx")

\#### sociodemographics model

ovitrap_model_2 <-

Ovitrap3<-Mosquitoes[complete.cases(Mosquitoes[ ,c("income_nmw", "secondary_education", "inhabitants", "rooms",

"ov_eggs","rain_month_c", "location_ov", "id_casa"

)]), ]

summary(Ovitrap3)

ovitrap_model2 <- glmer.nb(ov_eggs ~ income_nmw + secondary_education + inhabitants + rooms + rain_month_c +

location_ov +

(1 | id_casa), data = Ovitrap3)

summary_ovitrap_model2 <- summary(ovitrap_model2)

print(summary_ovitrap_model2)

library(car)

summarycar_ovitraps <- car::vif(ovitrap_model2)

print(summarycar_ovitraps)

options(na.action = "na.omit")

library(MuMIn)

options(na.action="na.fail")

step_ovitrap_model2<-dredge(ovitrap_model2) #### stepwise selection of variables for multivariable model

head(step_ovitrap_model2, 10)

\#### household characteristics model

ovitrap_house <-

Ovitrap4<-Mosquitoes[complete.cases(Mosquitoes[ ,c( "material_roof_ed","pavement", "yard", "window_screens", "flood_barriers",

"ov_eggs", "rain_month_c", "location_ov", "id_casa"

)]), ]

summary(Ovitrap4)

ovitrap_house <- glmer.nb(ov_eggs ~ material_roof_ed + pavement + yard + window_screens + flood_barriers + rain_month_c +

location_ov +

(1 | id_casa), data = Ovitrap4)

summary_ovitrap_house <- summary(ovitrap_house)

print(summary_ovitrap_house)

\### check multicollinearity

summarycar_ovitrap_house <- car::vif(ovitrap_house)

print(summarycar_ovitrap_house)

options(na.action = "na.omit")

options(na.action="na.fail")

step_ovitrap_house <-dredge(ovitrap_house) #### stepwise selection of variables for multivariable model

head(step_ovitrap_house, 10)

\### household practices model

ovitrap_practices <-

Ovitrap5<-Mosquitoes[complete.cases(Mosquitoes[ ,c("water_interruption", "plastic_containers", "flowerpots","other_container", "garbage_front",

"ov_eggs", "rain_month_c" , "location_ov", "id_casa"

)]), ]

summary(Ovitrap5)

ovitrap_practices <- glmer.nb(ov_eggs ~ water_interruption + plastic_containers + flowerpots + other_container + garbage_front + rain_month_c +

location_ov +

(1 | id_casa), data = Ovitrap5)

summary_ovitrap_practices <- summary(ovitrap_practices)

print(summary_ovitrap_practices)

\### check multicollinearity

summarycar_ovitrap_practices <- car::vif(ovitrap_practices)

print(summarycar_ovitrap_practices)

options(na.action = "na.omit")

library(MuMIn)

options(na.action="na.fail")

step_ovitrap_practices <-dredge(ovitrap_practices) #### stepwise selection of variables for multivariable model

head(step_ovitrap_practices, 10)

\### environmental model

options(na.action = "na.omit")

environmental_model <-

Ovitrap8<-Mosquitoes[complete.cases(Mosquitoes[ ,c("sewer_close","proportion_veg_30",

"ov_eggs", "rain_month_c", "location_ov", "id_casa"

)]), ]

summary(Ovitrap8)

environmental_model <- glmer.nb(ov_eggs ~ sewer_close + proportion_veg_30 + rain_month_c +

location_ov +

(1 | id_casa), data = Ovitrap8)

summary_environmental_model <- summary(environmental_model)

print(summary_environmental_model)

options(na.action = "na.omit")

\### check multicollinearity

summarycar_environmental_model <- car::vif(environmental_model)

library(MuMIn)

options(na.action="na.fail")

step_environmental_model<-dredge(environmental_model) #### stepwise selection of variables for multivariable model

head(step_environmental_model, 10)

\### Final model

options(na.action = "na.omit")

ovitrap_final_edit <-

Ovitrap9<-Mosquitoes[complete.cases(Mosquitoes[ ,c("plastic_containers","garbage_front","sewer_close","flood_barriers", "flowerpots", "material_roof_ed", "proportion_veg_30", "rain_month_c",

"ov_eggs", "location_ov", "id_casa"

)]), ]

summary(Ovitrap9)

\# Fit the final model

ovitrap_final_edit <- glmer.nb(ov_eggs ~ plastic_containers + garbage_front + sewer_close + flood_barriers + flowerpots + material_roof_ed + proportion_veg_30 + rain_month_c +

location_ov +

(1 | id_casa), data = Ovitrap9)

summary_ovitrap_final_edit <- summary(ovitrap_final_edit)

print(summary_ovitrap_final_edit)

options(na.action = "na.omit")

library(MuMIn)

options(na.action="na.fail")

step_ovitrap_final_edit<-dredge(ovitrap_final_edit) #### stepwise selection of variables for multivariable model

head(step_ovitrap_final_edit, 10)

\### the final global model without interaction term

options(na.action = "na.omit")

final_ovitrap_model_edit <-

Ovitrap10<-Mosquitoes[complete.cases(Mosquitoes[ ,c("plastic_containers","garbage_front","sewer_close", "rain_month_c",

"ov_eggs", "location_ov", "id_casa"

)]), ]

summary(Ovitrap10)

final_ovitrap_model_edit <- glmer.nb(ov_eggs ~ plastic_containers + garbage_front + sewer_close + rain_month_c +

location_ov + (1|id_casa), data = Ovitrap10)

summary_final_ovitrap_model_edit <- summary(final_ovitrap_model_edit)

print(summary_final_ovitrap_model_edit)

library(performance)

check_collinearity(final_ovitrap_model_edit)

check_overdispersion(final_ovitrap_model_edit)

\### the final model after accounting for ovitrap exposure duration

final_ovitrap_model_offset <- glmer.nb(

ov_eggs ~ plastic_containers + garbage_front + sewer_close +

rain_month_c + location_ov +

offset(log(days_ovi)) +	# <-- offset for trap duration

(1 | id_casa),

data = Ovitrap10

)

summary_final_ovitrap_model_offset<- summary(final_ovitrap_model_offset)

print(summary_final_ovitrap_model_offset)

check_collinearity(final_ovitrap_model_offset)

check_overdispersion(final_ovitrap_model_offset)

\# Extract fixed effects coefficients

fixed_effects <- fixef(final_ovitrap_model_edit)

\# Compute confidence intervals for fixed effects

confint_fixed_effects <- confint(final_ovitrap_model_edit, parm = "beta_")

\# Combine coefficients and confidence intervals

combined_fixed_effects <- cbind(Coef = fixed_effects, confint_fixed_effects)

\# Exponentiate the combined results

exp_combined_fixed_effects <- exp(combined_fixed_effects)

\# Print the exponentiated combined results

print(exp_combined_fixed_effects)

\### the final model with interaction term

options(na.action = "na.omit")

Ovitrap11 <- Mosquitoes[complete.cases(Mosquitoes[, c(

"plastic_containers","garbage_front","sewer_close",

"rain_month_c","ov_eggs","location_ov","id_casa","community","days_ovi"

)]), ]

final_ovitrap_model_interaction <- glmer.nb(

ov_eggs ~ plastic_containers + garbage_front + sewer_close*community +

rain_month_c + location_ov +

offset(log(days_ovi)) +	# offset for trap duration

(1 | id_casa),

data = Ovitrap11

)

summary(final_ovitrap_model_interaction)

fixed_effects_int <- fixef(final_ovitrap_model_interaction)

confint_fixed_effects_int <- confint(final_ovitrap_model_interaction, parm = "beta_")

combined_fixed_effects_int <- cbind(Coef = fixed_effects_int, confint_fixed_effects_int)

exp_combined_fixed_effects_int <- exp(combined_fixed_effects_int)

print(exp_combined_fixed_effects_int)
